# Responses to Environmental Enrichment Differ with Sex and Genotype in a Transgenic Mouse Model of Huntington's Disease

**DOI:** 10.1371/journal.pone.0009077

**Published:** 2010-02-12

**Authors:** Nigel I. Wood, Valentina Carta, Stefan Milde, Elizabeth A. Skillings, Catherine J. McAllister, Y.L. Mabel Ang, Alasdair Duguid, Nadeev Wijesuriya, Samira Mohd Afzal, Joe X. Fernandes, T.W. Leong, Jennifer Morton

**Affiliations:** Department of Pharmacology, University of Cambridge, Cambridge, United Kingdom; Emory University School of Medicine, United States of America

## Abstract

**Background:**

Environmental enrichment (EE) in laboratory animals improves neurological function and motor/cognitive performance, and is proposed as a strategy for treating neurodegenerative diseases. EE has been investigated in the R6/2 mouse model of Huntington's disease (HD), where increased social interaction, sensory stimulation, exploration, and physical activity improved survival. We have also shown previously that HD patients and R6/2 mice have disrupted circadian rhythms, treatment of which may improve cognition, general health, and survival.

**Methodology/Principal Findings:**

We examined the effects of EE on the behavioral phenotype and circadian activity of R6/2 mice. Our mice are typically housed in an “enriched” environment, so the EE that the mice received was in addition to these enhanced housing conditions. Mice were either kept in their home cages or exposed daily to the EE (a large playground box containing running wheels and other toys). The “home cage” and “playground” groups were subdivided into “handling” (stimulated throughout the experimental period) and “no-handling” groups. All mice were assessed for survival, body weight, and cognitive performance in the Morris water maze (MWM). Mice in the playground groups were more active throughout the enrichment period than home cage mice. Furthermore, R6/2 mice in the EE/no-handling groups had better survival than those in the home cage/no-handling groups. Sex differences were seen in response to EE. Handling was detrimental to R6/2 female mice, but EE increased the body weight of male R6/2 and WT mice in the handling group. EE combined with handling significantly improved MWM performance in female, but not male, R6/2 mice.

**Conclusions/Significance:**

We show that even when mice are living in an enriched home cage, further EE had beneficial effects. However, the improvements in cognition and survival vary with sex and genotype. These results indicate that EE may improve the quality of life of HD patients, but we suggest that EE as a therapy should be tailored to individuals.

## Introduction

HD is a genetic neurodegenerative disorder that is caused by an expanded CAG repeat in the coding region of the HD gene [1]. The disease is characterised by progressive striatal atrophy and the loss of neurons in frontal and temporal cortex, although by the end stages of the disease degeneration is also present in many subcortical regions. Patients with HD develop progressive motor, cognitive and psychological symptoms that invariably lead to death within 17–20 years after the onset of first symptoms.

There is no effective treatment available yet for HD. However, it has been shown that an active lifestyle (involving enhanced social, physical and mental components) protects against dementia and Alzheimer's disease in human patients (reviewed in [2]). It is thus possible that increased environmental stimulation of patients could be used to improve the symptoms and slow the progression of HD. Indeed, it has been suggested that physical, social and cognitive stimulation has beneficial effects in HD patients [3], [4].

To study the mechanisms by which lifestyle elements influence the progression of HD, aspects of an enhanced lifestyle can be mimicked in laboratory animals by EE. There were two aims of the current study. EE has been shown to have beneficial effects on the progression of motor symptoms and survival in the R6/1 and R6/2 mouse models of HD [5], [6], [7]. In these studies, EE was provided through multiple different forms of enhanced home cages, all of which had beneficial effects. An improved feeding regime, accompanied by regular behavioural testing, significantly enhanced the general well-being and life expectancy of R6/2 mice [5]. An enriched home cage delayed onset of motor symptoms, decreased severity of the clasping phenotype, and reduced the loss of peristriatal cerebral volume in R6/1 mice [6]. Even a low level of enrichment with food pellets and a cardboard tube placed in the home cage slowed the decline in rotarod performance in R6/2 mice [7], although a greater level of enrichment induced a marked improvement in rotarod tests, and delayed the loss of peristriatal cerebral volume in R6/2 brain [7].

To explain the beneficial effects of EE on motor and cognitive symptoms and survival of HD transgenic mice, several potential mechanisms have been suggested. For example, EE may cause an increase in striatal and hippocampal levels of brain-derived neurotrophic factor (BDNF; [8]), levels of which are known to be reduced in the striatum and hippocampus of HD mouse models [9] and in the *post mortem* brains of HD patients [10]. EE has also been found to specifically increase neurogenesis in the dentate gyrus of the hippocampus of a mouse model of HD [11]. Given that such changes in hippocampal function are possible, and that hippocampus-dependent learning is impaired in R6/2 mice [12], it is logical to expect that enrichment might have a beneficial effect on hippocampal learning and memory tasks. However, while evidence for improved spatial learning in response to EE has been found for the R6/1 model [13], [14], no such study has been conducted in R6/2 mice. For this reason, the first aim of the current study was to investigate the effects of EE on cognitive performance in the MWM task in R6/2 mice.

The second aim of the current study was to investigate the effects of sleep deprivation during circadian day. Disturbance of the sleep-wake cycle is commonly observed in HD patients and is mirrored by a progressive disintegration of circadian patterns of activity and a disruption of circadian clock gene expression in the suprachiasmatic nucleus (SCN) of R6/2 mice [15]. The treatment of R6/2 mice with the sedative drug Alprazolam, a therapy intended to restore their circadian rhythms, slowed cognitive decline, and improved clock-gene related functions [16]. Modulation of the sleep-wake cycle with Modafinil as well as Alprazolam also had beneficial effects on cognitive function and improved apathy [17]. This raises the possibility of an effective behavioural therapy involving sleep regulation to manage the symptoms of HD patients. Given that drug-induced wakefulness had beneficial effects on the cognitive performance of R6/2 mice [17], a period of continuous activity induced by enrichment might also impose sleep without the need for sedative drugs and have similar beneficial effects. The constraints of our animal facility mean that, in the current study, enrichment had to take place during circadian day. However, enrichment during circadian day might result in sleep deprivation and thus lead to deleterious effects. To test this possibility, we included groups in which we enforced wakefulness on mice, by physically handling these mice to keep them awake throughout the 6-hour daily period of experimentation.

## Materials and Methods

### Ethics Statement

All components of this study were carried out in accordance with the UK Animals (Scientific Procedures) Act, 1986, and with the approval of the University of Cambridge Licence Review Committee.

### Mice

Mice were taken from a colony of R6/2 transgenic mice [18] that is established in the Centre for Brain Repair, University of Cambridge, and maintained by backcrossing onto CBA x C57BL6 F1 female mice. Genotyping and CAG repeat length measurement were carried out by Laragen (Los Angeles, CA, USA). The number of CAG repeats of R6/2 mice used in this study (N = 80) was 249±0.6 (mean ± SEM) as determined by GeneMapper (note that the CAG repeat number measured by GeneMapper differs from that measured by sequencing. To convert the CAG repeat numbers determined by GeneMapper technique to the CAG repeat number determined by sequencing technique (which more closely represents the true CAG repeat number) the following formula needs to be applied: SEQ CAG no. (true CAG no.) = 1.0427 * GM CAG no. + 1.1695; personal communication, Dr J. Li, Laragen).

Mice were kept in home cages comprising single sex, single genotype groups of 10. Our mice live as standard in an enhanced environment with increased amounts of bedding and nesting materials, and additional hydrated food (see below). Clean cages were provided twice weekly, with grade 8/10-corncob bedding, finely shredded paper for nesting, and a red plastic nest box. The mice were maintained on a 12 hour light: 12 hour dark cycle, at a temperature of 21–23°C and a humidity of 55±10%. The mice had *ad libitum* access to water (using water bottles with elongated spouts) and standard dry laboratory food (RM3(E) rodent pellets, Special Diet Services, Witham, UK). In addition, once a day, a mash was prepared by soaking 100 g dry food in 230 ml water until the pellets were soft and fully expanded. The mash was placed on the cage floor, improving access to food and water for the R6/2 transgenic mice. This feeding regime has been shown previously to be beneficial [5].

### Environmental Enrichment

The mice were tested in four different groups with variations in environmental enrichment conditions:

- Home cage/no handling
- Home cage/handling
- Playground/no handling
- Playground/handling

**Figure 1:**
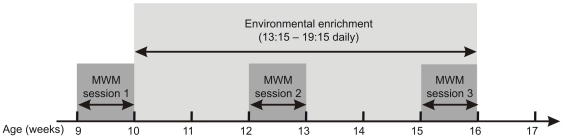
Experimental timeline for enrichment and testing. Baseline data in the MWM were obtained at 9 weeks of age. Further MWM training and testing were performed at 12 and 15 weeks of age. Environmental enrichment took place between 10 to 16 weeks of age.

Each group comprised four cages, containing male WT (n = 10), female WT (n = 10), male R6/2 (n = 10) and female R6/2 (n = 10) mice. The timeline for testing and treatments for the whole experiment is shown in Figure 1.

Mice in the home cage/no handling groups were confined to their home cages without additional enrichment throughout the whole experiment. EE was provided to the other groups of mice from 10 to 16 weeks of age for 6 hours each day (13∶15h to 19∶15h). Enrichment was administered as either access to playgrounds or through gentle handling. The playgrounds consisted of large Perspex™ boxes (60×30×45 cm) containing ropes, ladders, running wheels and toys (Movie S1). The toys and their configuration were changed daily to maintain the element of novelty, maximising the stimulating nature of the environment. After each session, playground cages and toys were cleaned using 1% acetic acid. Handling involved gentle manipulation of the mice if they spent longer than 60 seconds immobile throughout the period of enrichment (Movie S2).

### Behavior and Handling Data Collection

Records were kept throughout the daily experimental period (1315h to 1915h) of the handling given and also of the behavioural activity of the mice. For the groups receiving handling, individual records were kept of every occasion on which each mouse required handling. The behaviour of each mouse in every cage was recorded by a trained observer every 15 minutes. Three or 4 observers were randomly assigned to observe the mice during a specific 2-hour time slot within the daily experimental period (6 hours). Due to the overt phenotype of the R6/2 mice, observers could not be blind to the genotype of the mice. The observed behaviours were classified as “active” (score 1) or “inactive” (score 0) and these scores were used for quantitative analysis of behaviour.

### Body Weight and Survival Analysis

Body weight of all mice was recorded twice weekly from the start of treatment (age 9 weeks) throughout the experiment (6 weeks) and after that until death (for R6/2 mice) or 27 weeks of age (for WT mice). Note that we have only presented R6/2 weights up to 19 weeks, since beyond this age mice started to reach the end-stage of the phenotype, and were killed for humane reasons. We have shown in previous studies that mice that lose the most body weight tend to die first, and that this distorts weight data [19]. Therefore, statistical analysis was conducted on R6/2 weights between the ages of 9–19 weeks while data from 9–27 weeks of age was used for the analysis of WT weights.

Age of death was recorded for all R6/2 mice. Mice were killed if they were moribund, or lacked a righting reflex, or failed to rouse for their mash, or did not respond to gentle stimulation.

### Morris Water Maze Task

Spatial learning was tested using the protocol as described in Wood *et al.* [20]. Briefly, a circular white plastic pool 120 cm in diameter and 50 cm in height was filled to a depth of 30 cm with water and maintained at 23°C. A small quantity of non-toxic white paint was added to render the water opaque. Four positions around the edge of the tank were arbitrarily designated as N, S, E, and W, providing four alternative start positions and dividing the tank into four quadrants: NE, SE, SW and NW. A circular clear Perspex platform of 10 cm diameter was placed at the midpoint of one of the four quadrants and submerged 0.5 cm below the water surface. Extramaze cues were minimised by placing screens around the tank. Various visible cues were added to the screens to aid spatial discrimination. Mice were tracked in the maze using the HVS tracker system (HVS Image 2020, Hampton, UK). During training, mice received four trials per day with an inter-trial interval of 10–15 minutes. Each mouse was placed in the pool facing the wall at one of four pseudorandomly chosen starting positions (N, S, E, W), and allowed to swim until it located and climbed onto the submerged platform. Any mouse that failed to locate the platform within 60 seconds was placed on the platform by hand. All mice remained on the platform for 15 seconds, after which they were briefly dried with paper tissues before being returned to a cage containing clean shredded paper bedding, and warmed by a heating lamp. On completion of all four trials, the mice were dried thoroughly and returned to their home cages. A probe trial consisted of a single 60 second trial with the platform removed. After 60 seconds the mice were removed to a drying cage as before.

Baseline data were obtained from the mice at 9 weeks of age in the MWM (see Figure 1) before they were assigned to their experimental groups. Mice were tested under a standard protocol (6 days training, with 4 trials a day, to a single platform position, followed on day 7 by a single probe trial). After a two week period of EE, mice received a single probe trial (retention), followed by five days of training (4 trials per day) to the same platform position as in the baseline test and a single probe trial (re-acquisition). After a further two weeks of enrichment, mice underwent a third session in the MWM. This took the same form as the second session (single probe trial, then five days of training, then a further single probe trial). During the second and third MWM sessions, mice continued to be exposed to their respective enrichment condition. Training or testing in the MWM took place in the morning while enrichment was provided in the second half of the light period (13∶15–19∶15 hrs) as described above. After the end of the last MWM session at 16 weeks of age, mice were returned to their home cages.

### Data Analysis

Performance in the probe trials was evaluated by measuring the time spent in virtual quadrants and zones in the water maze, proximity to the platform location and swim speed. For statistical analysis of percent time spent in quadrants and zones of the water maze, a five-way repeated measures ANOVA was used (factors: sex, genotype, handling/no handling, home cage/playground and quadrant or zone number). For statistical analysis of proximity and swim speed we used a four-way repeated measures ANOVA (factors: sex, genotype, handling/no handling, home cage/playground). Group comparisons were made using Sidak- adjusted multiple comparisons. Behavioural scores reflecting activity of the mice during the experimental period were analysed using 4-way repeated measures ANOVA as above. The number of handling events was analysed using three-way repeated measures ANOVA (factors: sex, genotype, playground/home cage) with subsequent Sidak-adjusted multiple comparisons. The body weight data for WT and R6/2 mice were analysed in separate three-way repeated measures ANOVA, (factors: sex, playground/home cage and handling/no handling). Survival data were analysed using a log rank test.

Statistical analyses were performed using SPSS Statistics 17.0 (SPSS Inc., Chicago, USA) or Prism 4 (GraphPad Software Inc., San Diego, USA).

## Results

### Morris Water Maze

**Figure 2:**
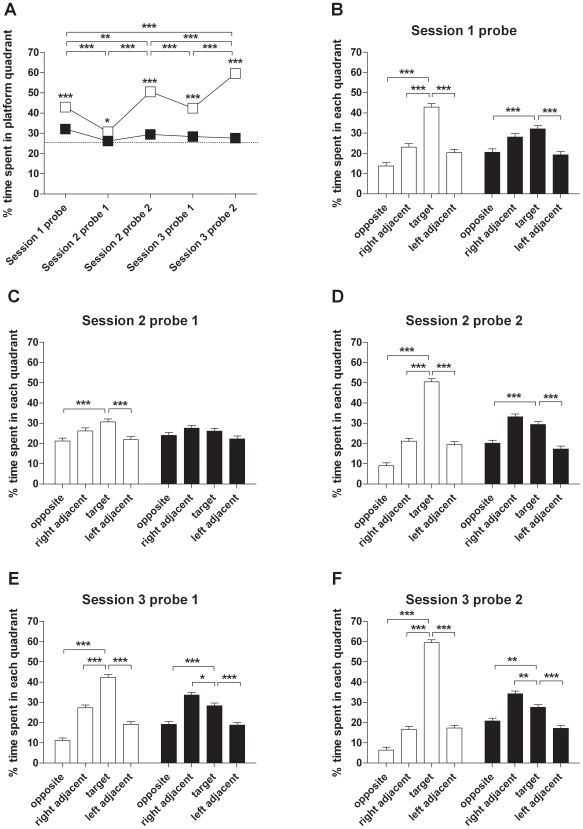
Quadrant preference of mice during Morris water maze probe trials. Percent time spent in the target quadrant in all probe trials is shown for WT and R6/2 mice (A). Comparisons of percent time spent in all quadrants are shown for WT and R6/2 mice for all MWM probe trials (B-F). Note that data from both sexes and all experimental groups were pooled for each genotype. All data shown are means ± s.e.m. Where error bars are not visible, they are obscured by symbols. * p<0.05, ** p<0.01, *** p<0.001.

Traditional measures of performance in MWM probe trials include percent times spent in the target quadrant and zone. Analysis of the percent time spent in the target quadrant revealed a main effect of genotype (F_(1,141)_ = 214.113, p<0.001). In all probe trials, WT mice spent significantly more time in the target quadrant than R6/2 mice (Figure 2A). There were no main effects of sex, handling or playground exposure, either beneficial or deleterious (not shown). This suggests that enrichment had no influence on the performance of WT or R6/2 mice in the MWM as measured by the time spent in the target quadrant. Thus, for presentation, data from both sexes and all enrichment groups has been combined for presentation (Figure 2). With the exception of the probe trial after the first retention interval (session 2 probe 1), WT mice displayed a significant preference for the target quadrant in every probe trial (Figure 2B-F). This indicates learning of the platform position as no preference for a particular quadrant was observed in either WT or R6/2 mice when they were first exposed to the MWM (Figure S1A). No preference for the target quadrant was observed in R6/2 mice in any of the probe trials (Figure 2B-F). Interestingly, in the third MWM session, R6/2 mice showed a significant preference for the quadrant right-adjacent to the target quadrant (Figure 1E, F). Mice were placed into the pool from a random starting position. However, the right-adjacent to the target quadrant is the quadrant from which the experimenter approached to remove the mice from the pool after a trial, or to guide them to the platform position during training. This suggests some ability for spatial learning in the R6/2 mice, but they used a strategy that the MWM is not designed to test.

**Figure 3:**
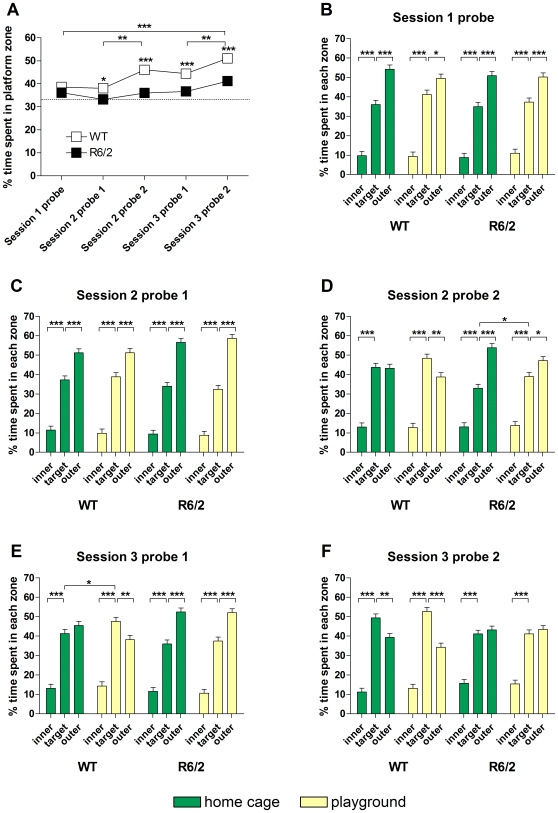
Zone preference of mice during Morris water maze probe trials. Percent time spent in the target zone in all probe trials is shown for WT and R6/2 mice (A). Data from both sexes and all experimental groups were pooled for each genotype. Comparisons of percent time spent in all quadrants are shown for WT and R6/2 mice from home cage and playground groups for all MWM probe trials (B-F). Data from both sexes as well as handling and no handling groups were pooled. All data shown are means ± s.e.m. Where error bars are not visible, they are obscured by symbols. * p<0.05, ** p<0.01, *** p<0.001.

In an analysis of the percent time spent in the target zone there was a main effect of genotype (F_(1,136)_ = 34.933, p<0.001). No main effects were found for sex or handling (not shown) and so data from both sexes and handling conditions were combined for presentation in Figure 3. There was, however, a main effect of playground exposure (F_(2,405)_ = 5.738, p = 0.003). *Post hoc* analysis revealed that this effect was present in WT (p = 0.014) but not R6/2 mice (p = 0.309), suggesting a beneficial effect of enrichment on the zone preference of WT mice. With the exception of the first probe trial, WT mice spent significantly more time in the target zone than R6/2 mice (Figure 3A). In the probe trials after training in the first MWM session and after the retention interval before the second training session, neither WT nor R6/2 mice showed a preference for the target zone, irrespective of their enrichment condition (Figure 3B, C). The preference for the outer zone of the MWM observed in these trials in all groups of mice reflects the initial response to the MWM that was also present in the first training trial of the first MWM session (Figure S1B). During training in the second MWM session, WT playground mice developed a preference for the target zone while WT home cage mice did not differentiate between the outer and the target zones (Figure 3D). The same difference between home cage and playground WT mice was found after the retention interval before the third MWM session (Figure 3E). After training in the third MWM session, both WT groups showed significant preference for the target zone (Figure 3F ).Throughout all training sessions, R6/2 mice never showed a preference for the target zone (Figure 3A-F). Overall, data from the analyses of percent times spent in the target quadrant and zone during probe trials suggests that WT mice successfully learned the platform position while R6/2 mice consistently failed to do so. While enrichment by playground exposure did not affect the MWM performance of R6/2 mice, WT playground mice showed a faster learning of the correct MWM zone than WT home cage mice. However, we found no effect of handling on either WT or R6/2 mice.

**Figure 4:**
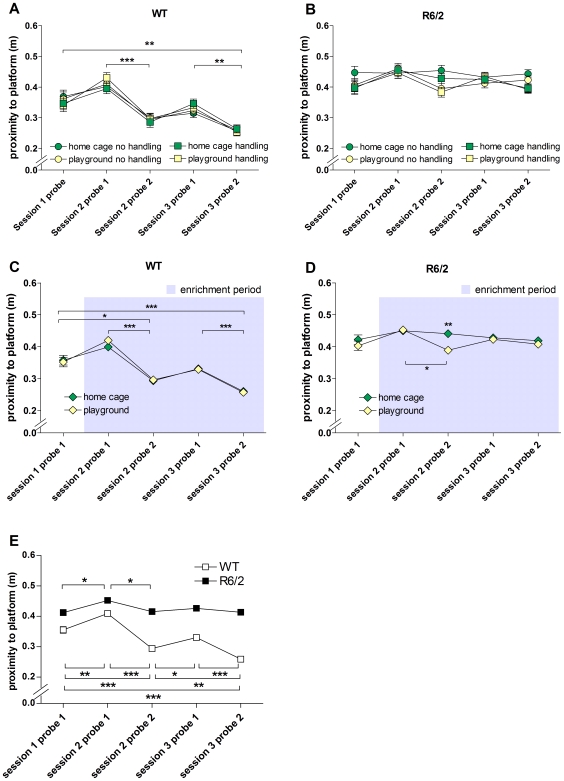
Proximity of mice to platform position during Morris water maze probe trials. Proximity to platform position for home cage/no handling, home cage/handling, playground/no handling and playground/handling groups of WT (A) and R6/2 (B) mice in all MWM sessions, with data from both sexes pooled. Data from handling and no handling groups as well as from both sexes were pooled to compare home cage and playground groups of WT (C) and R6/2 (D) mice. Data from both sexes and all experimental groups were pooled for WT and R6/2 mice (E). Grey shading in C, D indicates period of enrichment. All data shown are means ± s.e.m. Where error bars are not visible, they are obscured by symbols. n.s. non-significant, * p<0.05, ** p<0.01, *** p<0.001.

Recent work has suggested that the proximity score (also known as the Gallagher score) may be a more sensitive measure of performance than these classical parameters [21]. Therefore, we also measured proximity to the platform position in the probe trials. An analysis of data from all probe trials revealed no main effect of sex. Therefore, data from both sexes were combined for the purpose of presentation (Figure 4). There was a main effect of genotype. WT mice in all enrichment groups improved their performance during the course of the experiment by swimming closer to the original platform position (F_(4,135)_ = 61.459, p<0.001; Figure 4A). R6/2 mice, in contrast, showed no change in proximity throughout the experiment, suggesting that the R6/2 mice were impaired in learning the task (Figure 4B). This combined analysis revealed no overall effect of enrichment in playgrounds on MWM performance in either WT (Figure 4C) or R6/2 mice (Figure 4D), suggesting that EE had no effect on the rate of learning of these mice. Throughout the experiment, WT mice performed better than R6/2 mice, irrespective of sex or enrichment (F_(1,138)_ = 224.657, p<0.001; Figure 1E).

**Figure 5:**
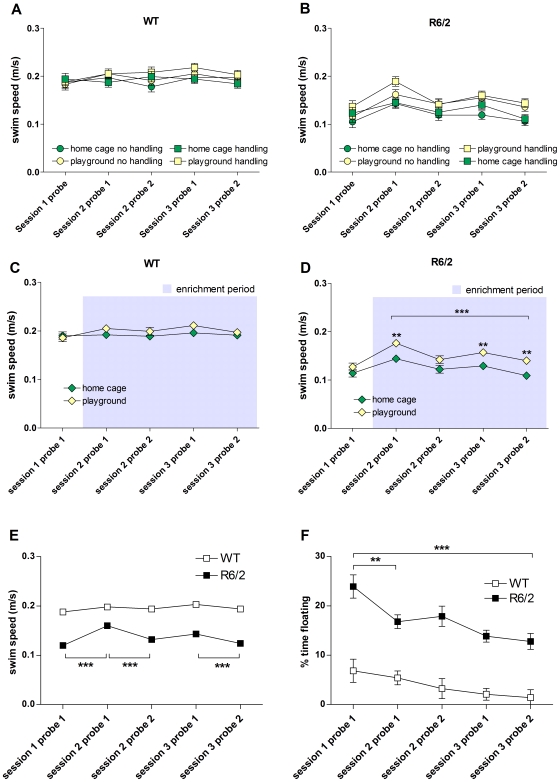
Swim speed of mice during Morris water maze probe trials. Swim speed for home cage/no handling, home cage/handling, playground/no handling and playground/handling groups of WT (A) and R6/2 (B) mice in all MWM sessions, with data from both sexes pooled. Data from handling and no handling groups as well as from both sexes were pooled to compare home cage and playground groups of WT (C) and R6/2 (D) mice. Data from both sexes and all experimental groups were pooled for WT and R6/2 mice and are shown in (E). The percentage of time spent floating in each MWM trial is shown for WT and R6/2 mice, with data from both sexes and all experimental groups pooled for each genotype (F). Grey shading in C, D indicates period of enrichment. All data shown are means ± s.e.m. Where error bars are not visible, they are obscured by symbols. n.s. non-significant, * p<0.05, ** p<0.01, *** p<0.001.

We measured swim speed in the MWM, as an index of motor performance. Neither WT nor R6/2 mice showed any changes in swim speed over time (Figure 5A, B). An analysis of main effects showed that playground mice were faster swimmers than home cage mice (F_(1,141)_ = 8.100, p = 0.005). This effect was evident in R6/2 mice from the first probe trial after the start of enrichment and onwards (F_(1,141)_ = 11.947, p<0.001; Figure 5D) but was not seen in WT mice (Figure 5C). However, it was also found that, as expected, R6/2 mice were consistently slower swimmers than WT mice (F_(1,141)_ = 105.580, p<0.001; Figure 5E). During each probe trial, R6/2 mice spent more time floating than WT mice (F_(1,141)_ = 45.510, p<0.001; Figure 5F). There was, however, a significant decrease in time spent floating by R6/2 mice over the course of the experiment (p<0.001), suggesting that floating was not a strategy adopted by R6/2 mice because their phenotype made them too weak to swim.

**Figure 6:**
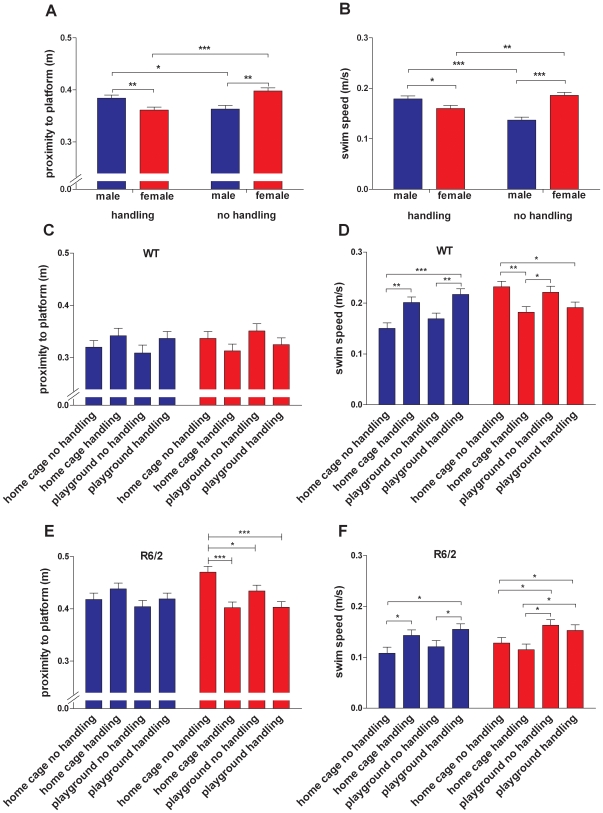
Sex and handling interaction on Morris maze performance and swim speed. Platform proximity (A) and swim speed (B) data were combined across all probe trials. Data are shown for male (blue bars) and female (red bars) groups for ‘handling’ and ‘no handling’ conditions with data from both genotypes as well as home cage and playgrounds groups pooled. Proximity to the platform position and swim speed combined across probe trials are shown for all WT (C, D) and R6/2 (E, F) groups. Data for male and female mice are shown separately. All data shown are means ± s.e.m. n.s. non-significant, * p<0.05, ** p<0.01, *** p<0.001.

Interestingly, when data from probe trials were pooled throughout the experiment for analyses of main effects, we found a significant interaction between sex and handling for both platform proximity (F_(1,141)_ = 21.540, p<0.001) and swim speed (F_(1,141)_ = 34.087, p<0.001). Handling had a beneficial effect on the performance of female mice (p<0.001), but a detrimental effect on the performance of males (p = 0.012; Figure 6A), irrespective of genotype or playground exposure. Similarly, handling reduced swim speed in female mice (p = 0.002), but increased it in male mice (p<0.001; Figure 6B). *Post hoc* analyses revealed further sex-specific effects of enrichment. Female R6/2 mice from all enrichment groups showed better awareness of the platform position than the home cage/no handling mice (home cage/handling: p<0.001, playground/no handling: p = 0.03, playground/handling: p<0.001; Figure 6E). This effect was not present in male R6/2 (Figure 6E) or WT mice of either sex (Figure 6C). Thus, data from the proximity analysis suggest that enrichment through access to playgrounds or handling improves cognitive performance in female mice only. A sex–specific effect of the enrichment conditions was also found for swim speed in the MWM, where both WT and R6/2 male mice increased their swim speed in response to handling. This was found for the home cage (WT, p = 0.001; R6/2, p = 0.041; Figure 6D, F) as well as for the playground groups (WT, p = 0.004; R6/2, p = 0.044; Figure 6D, F). Playground exposure without handling did not lead to significant increases in swim speed of male mice compared to home cage/no handling groups. Compared to male mice of both genotypes, R6/2 females exhibited the opposite response to enrichment conditions. They showed an increase in swim speed in response to the playground condition but not in response to handling. The positive effect of playground exposure on swim speed of R6/2 females was found for both no handling (p = 0.034) and handling groups (p = 0.018; Figure 6F). WT female mice responded to handling with a decrease in swim speed as compared to the home cage/no handling group, both in the home cage/handling (p = 0.002) and playground/handling groups (p = 0.010; Figure 6D). As the playground/no handling WT females did not show a reduced swim speed compared to the home cage/no handling group, this suggests that the effect was specific for handling and does not apply to all forms of enrichment (Figure 6D). Overall, the swim speed data suggest sex and genotype–specific responses of mice to the enrichment conditions.

### Daytime Activity

**Figure 7:**
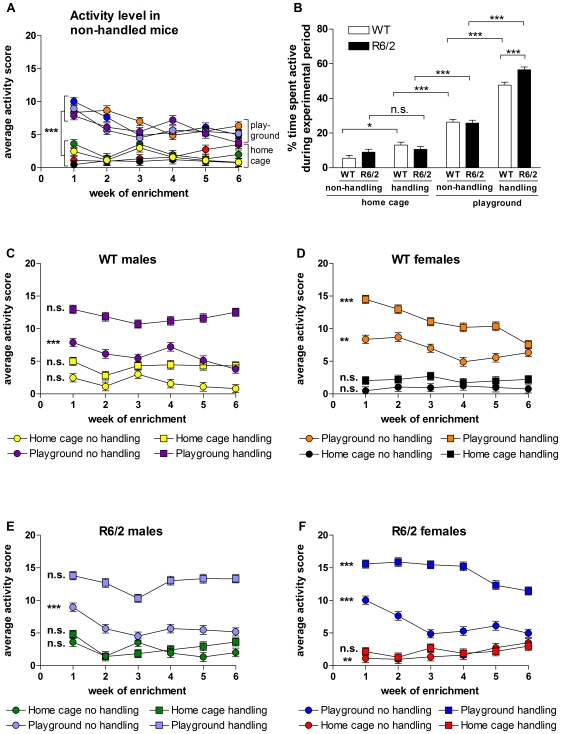
Activity of mice during the enrichment period. Average daily activity scores are shown for weeks 1 to 6 of the experiment for all no handling groups (A). Average activity scores throughout the whole experiment are presented for all groups of WT and R6/2 mice in (B), with data from male and female mice pooled. Average daily activity scores in weeks 1 to 6 of the experiment are shown for all WT male (C), WT female (D), R6/2 male (E) and R6/2 female (F) groups. For key to symbols in (A), see (C), (D), (E) and (F). Symbols left of data series indicate significance of decline from week 1 to week 6. All data shown are mean ± s.e.m. Where error bars are not visible, they are obscured by symbols. n.s. non-significant, * p<0.05, ** p<0.01, *** p<0.001.

The effect of enrichment on daytime activity measured during the daily experimental period in the mice is shown in Figure 7. The behaviour of each mouse was scored as “active” or “inactive” in 15-minute intervals. Data were analysed on a day-by-day basis, but for clarity only weekly averages are presented. In the no handling groups, there were significantly higher levels of activity in the playground groups compared to the home cage groups, irrespective of genotype and sex (F_(1,143)_ = 125.438, p<0.001; Figure 7A). This suggests strongly that the playgrounds were intrinsically stimulating, and remained so throughout the enrichment period. Overall, activity of the mice was lowest in the home cage/no handling groups, and highest in the playground/handling group, regardless of genotype (Figure 7B). In the playground/handling groups, R6/2 mice were more active than WT mice (p<0.001; Figure 7B). Although the playgrounds stimulated activity in the mice, the playground/no handling groups showed the greatest decline in activity between weeks 1 and 6 of the enrichment period. This was true for WT male (p<0.001; Figure 7C), WT female (p = 0.008; Figure 7D), R6/2 male (p<0.001; Figure 7E) and R6/2 female (p<0.001; Figure 7F) mice. Female mice in the playground/handling groups also showed a decline in activity during the enrichment period (p<0.001; Figure 7D, F), an effect that was not seen in male mice (Figure 7C, E).

### Handling

**Figure 8:**
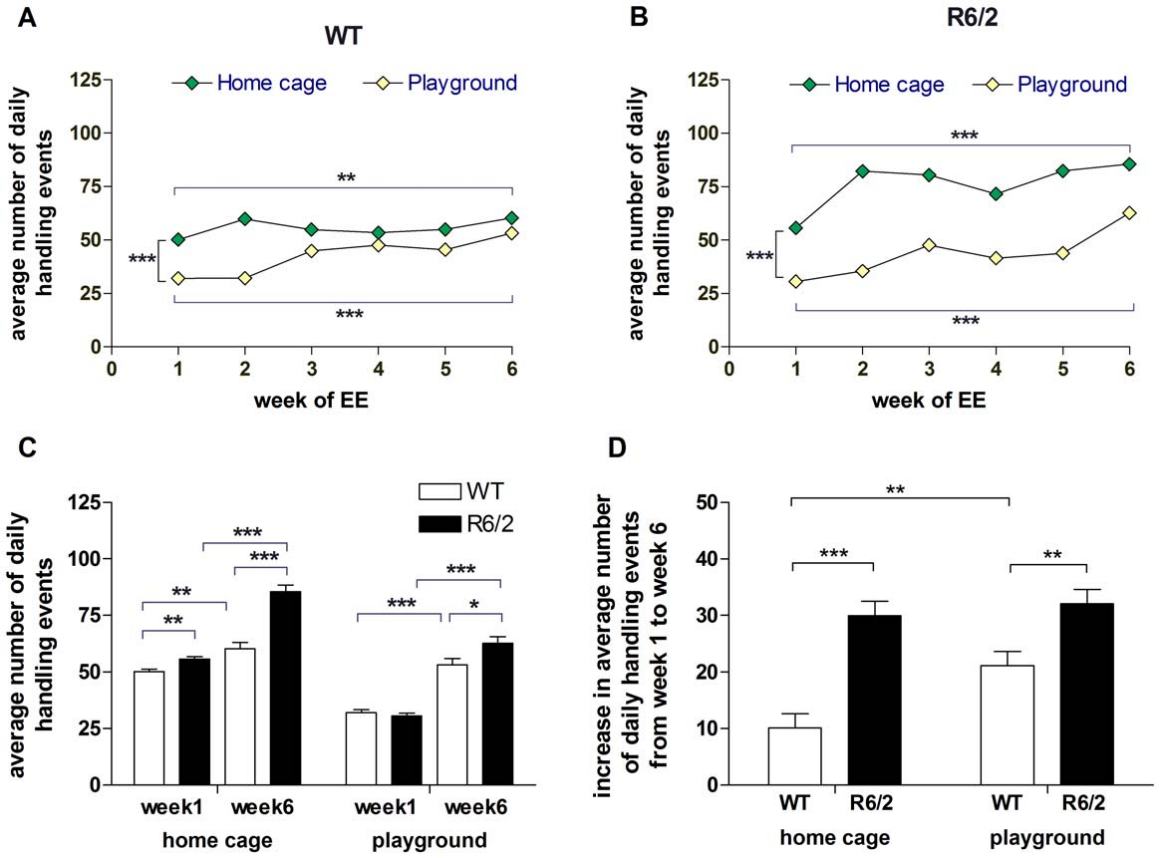
Amount of handling needed to keep the mice awake during the enrichment period. The average number of daily handling events required by each mouse during weeks 1 to 6 of the experiment for home cage and playground groups in WT (A) and R6/2 (B) mice. Some of these data are reproduced in (C) to allow comparisons between WT and R6/2 groups in home cage and playground conditions for the first and last weeks of the experiment. (D) shows the increase in the number of daily handling events from week 1 to week 6 of the experiment that were required by WT and R6/2 mice in home cage and playground groups. All data shown are means ± s.e.m. Where error bars are not visible, they are obscured by symbols. n.s. non-significant, * p<0.05, ** p<0.01, *** p<0.001.

We examined the amount of handling that was needed to keep the mice awake during the enrichment period. As expected, home cage mice needed more handling than playground mice, whether WT (F_(1,72)_ = 27.618 p<0.001; Figure 8A) or R6/2 (F_(1,72)_ = 172.766, p<0.001; Figure 8B). All mice needed more handling at the end of the experiment than the beginning, regardless of the playground enrichment condition (WT home cage, p = 0.002; WT playground, p<0.001; R6/2 home cage, p<0.001; R6/2 playground, p<0.001: Figure 8A, B). To compare the increase in handling required over the course of the experiment, data from the first (week 1) and the last (week 6) week of the experiment were analysed (Figure 8C, D). R6/2 home cage mice needed more handling than WT home cage mice both in week 1 (p = 0.002) and week 6 (p<0.001; Figure 8C) of the experiment. In the playground groups, this genotype difference was not present in week 1 but was observed in week 6 (p = 0.021; Figure 8C). Although all groups needed significantly more handling in week 6 than in week 1 of the experiment, the need for handling increased more strongly in R6/2 than in WT mice for both home cage (p<0.001) and playground groups (p = 0.003; Figure 8D). There was no difference in the increase in handling needed between R6/2 groups, but WT playground mice showed a greater increase in handling needed than the home cage group (p = 0.003, Figure 8D).

### Body Weight

**Figure 9:**
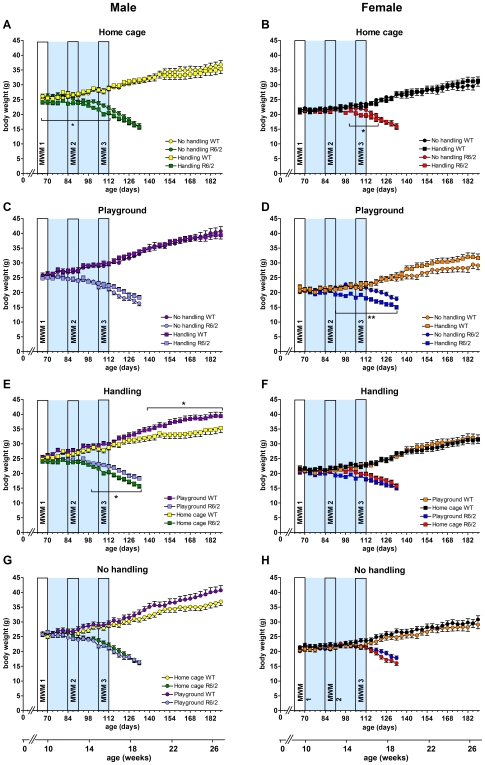
Body weights. Body weights were measured from 9.5 to 19 weeks for R6/2 and 9.5 to 27 weeks for WT groups. Data are separated by sex, and shown for home cage (A, B), playground (C, D), handling (E, F) and no handling (G, H) conditions. MWM1, 2 and 3 are periods of Morris water maze testing. Grey shaded areas represent the enrichment period. All data shown are mean ± s.e.m. Where error bars are not visible, they are obscured by symbols. n.s. non-significant, * p<0.05, ** p<0.01, *** p<0.001.

Body weights of the mice were recorded until the last R6/2 mouse was killed due to ill health at 27 weeks of age (Figure 6). Data are presented up to 19 weeks for R6/2 mice, as beyond this point the drop out of mice made the data difficult to analyse. Data for WT mice are shown up to 27 weeks, when the experiment finished. Analysis revealed the expected main effect of sex, with males being heavier (F_(1,134)_ = 159.148, p<0.001).There was also an expected main effect of genotype as the R6/2 mice started to lose weight from around 12 weeks of age. Group comparisons revealed differences in the female R6/2 home cage (between 14.5 and 17 weeks of age) and playground groups (13.5 to 19 weeks of age), where handling significantly reduced body weights compared to the no handling group (home cage: p = 0.042, playground: p = 0.06 Figure 9B, D). A similar negative effect of handling on body weight for was found for male R6/2 mice in the home cage groups between 9 and 16 weeks of age (p = 0.024; Figure 9A) but not in the playground groups (Figure 9C). In the R6/2 male handling groups, playground exposure led to an increase in body weight between 14.5 and 19 weeks of age (Figure 9E). There were no differences between any of the WT groups except in the male handling groups, where the playground mice increased weight significantly compared to the home cage mice, from 22 weeks onwards (p = 0.027; Figure 9E). Although a similar tendency was found for the male no handling groups, it did not reach statistical significance (p = 0.096; Figure 9G).

### Survival

We looked at the effect of EE on survival in R6/2 mice. Median survival for all groups is presented in Table 1. In the home cage groups, handling had no effect on the age at death in male mice, but had a beneficial effect in female mice (p = 0.001; Table 2). In the playground groups, handling had a detrimental effect on survival in male mice (p<0.001; Table 3), but no effect in female mice (Table 2). Both male and female playground/no handling mice lived significantly longer than those in the home cage/no handling groups (male: p = 0.002, female: p = 0.011; Table 2, 3). In the mice that were handled, there was no difference in survival between home cage and playground groups of either sex (Table 2, 3).

**Table 1:** Median survival times of R6/2 mice.

		Median survival (days)	
Sex	Group	No handling	Handling
Male	Home cage	141	156
	Playground	163	152
Female	Home cage	156	177
	Playground	176	158

**Table 2:** Survival comparisons using log-rank test between groups of female R6/2 mice.

	Home cage/no handling	Home cage/handling	Playground/no handling	Playground/handling
Home cage/no handling	x	**	*	x
Home cage/handling	**	x	x	n.s.
Playground/no handling	*	x	x	n.s.
Playground/handling	x	n.s.	n.s.	x

*p<0.05, ** p<0.01, *** p<0.001, n.s. not significant, x comparison not valid.

**Table 3:** Survival comparisons using log-rank test between groups of male R6/2 mice.

	Home cage/no handling	Home cage/handling	Playground/no handling	Playground/handling
Home cage/no handling	x	n.s.	**	x
Home cage/handling	n.s.	x	x	n.s.
Playground/no handling	**	x	x	***
Playground/handling	x	n.s.	***	x

*p<0.05, ** p<0.01, *** p<0.001, n.s. not significant, x comparison not valid.

## Discussion

Since EE was first shown to improve survival in the R6/2 mouse model of HD [5], numerous studies have been conducted to further investigate its effects in models of neurodegenerative disease [7], [8], [13], [14], [22]. Most of these studies have used the R6/1 mouse, which has a repeat length of approximately 115 CAG repeats, with a delayed onset of, and less severe, phenotype than the R6/2 mouse. Studies using EE in the R6/2 mouse are far fewer, because the early onset and severity of the phenotype makes it much more challenging to show beneficial effects. However, as we have already had success in improving the lifespan of these mice through home cage enrichment [5], we wanted to see whether we could also improve the cognitive dysfunction in R6/2 mice through access to additional enrichment.

In this study, we examined the effect of two different types of EE on the cognitive function, body weight and survival in R6/2 mice. We found a range of effects, both beneficial and detrimental, with significant genotype and sex effects. First, we assessed cognitive performance in probe trials in the WT task using the classical methods of percent time spent in the target quadrant or zone. These showed the expected deficits in R6/2 as compared to WT mice, and an absence of any beneficial (or detrimental) effects arising from enrichment in the R6/2 mice. The Gallagher proximity score has been demonstrated to be more sensitive for detecting group differences than these traditional measures [21]. However, we again found no improvements over time in the R6/2 groups. The most likely explanation for this finding is that the deficit in spatial learning apparent by 9 weeks of age, when MWM training in the current study began, cannot be reversed by means of EE. It might be worthwhile to begin both enrichment and cognitive testing at an earlier age, to see if EE can prevent the development of cognitive deficits, as has been shown in R6/1 mice [14]. However, as R6/2 mice have been shown to have deficits in the MWM from as early as 3.5 weeks of age [12], this may not be possible.

Although EE did not improve the overall cognitive performance of R6/2 mice in the MWM during the course of the study, it did produce significant differences between groups. For example, all mice in enriched female R6/2 groups performed significantly better than those in the home cage/no handling group. This suggests that female R6/2 mice may be more sensitive to the beneficial effects of EE than the other groups. This suggestion is reinforced by the finding that handling had beneficial effects on the cognitive performance of female mice of both genotypes, while having a negative effect in male mice. Interestingly, we found the reverse effect with regard to swim speed, where handling produced an increase in swim speed in male mice, but a decrease in females. While this increase in swim speed in males might at face value suggest a beneficial effect, it may also be a response to increased stress caused by handling, since it has been shown that stress in rats and mice can cause an increase in swim speed in the MWM [23], [24]. It should be noted that stress in rats also caused a deficit in MWM probe trial performance [25]. In addition, it has been reported that a stress paradigm had opposite effects on MWM performance of male and female rats, with female rats deriving a beneficial effect while males suffered detrimental effects [26]. It is possible that, in the male R6/2 mice, the lack of effect of enrichment on proximity score, combined with increased swim speed, is indicative of sex-specific increased stress caused by handling. It would be interesting, in future experiments, to measure levels of circulating corticosterone or testosterone to explore this possibility further.

In order to keep the playgrounds as stimulating as possible, we changed some of the toys every day to maintain an element of novelty. This appeared to have the desired effect, as mice in the playground/no handling groups were more active than mice in the home cage/no handling groups throughout the experiment. We found a graded effect of activity across the groups, with the least active mice being the home cage/no handling groups, followed by the home cage/handling, playground/no handling, and the most active being the playground/handling groups. Notably, although the playground groups were more active throughout the entire experiment, the playground/no handling groups showed the greatest decline in activity between weeks 1 and 6. While this is in part due to the fact that they were very active to start with and so had further to decline, it does seem that the stimulatory effect of the playgrounds was falling by the end of the study. In addition to the decline in activity seen in all of the playground/no handling groups, the female playground/handling groups also showed a significant decline in activity between weeks 1 and 6, which strongly suggests that even when the contents of the playgrounds were regularly changed, female mice habituated to the playgrounds faster than male mice. This adds more weight to the idea that there are significant differences between the sexes in the way that they respond to EE, and that some forms of enrichment may be more beneficial to one sex than the other. Similar sex – specific effects of EE on MWM learning and memory have previously been reported for a mouse model of Down syndrome where EE had beneficial effects on spatial learning in female mice but deleterious effects in male mice [27].

The possibility that the stimulatory effect of the playgrounds was declining over the course of the experiment is further supported by the finding that, even though the playground mice were more active and required less handling than home cage mice, by the end of the study the amount of handling they needed had increased significantly. The R6/2 mice needed a larger increase in handling between weeks 1 and 6 than WT mice to keep them active during the enrichment period, in both the home cages and playgrounds. This correlated with the onset of an overt phenotype in the R6/2 mice, although the mice were still capable of climbing and running. It is possible that as part of their developing phenotype with increasing age, R6/2 mice find their surroundings less interesting than do their WT littermates and display a reduction in voluntary activity. This could reflect an element of apathy. Apathy has been shown to be a major component of the disease in patients [28], and it becomes more severe with illness duration, and motor and cognitive dysfunction [29], [30], [31]. There are currently no reliable tests for apathy in rodents, but an apathy-like syndrome has been identified and successfully treated in R6/2 mice [17]. It would be interesting to apply the same pharmacological intervention to an EE study, to see whether improving the circadian rhythm has added benefits in enriched mice.

The need for an increased amount of handling to keep the R6/2 mice active as the experiment progressed may be due to a developing dysfunction in their circadian rhythms. We have shown that as R6/2 mice age, their circadian activity changes from a pattern of discrete extended periods of activity and sleep, to a constant level of very short periods of activity and inactivity [15]. It is not clear what causes this change. The main mammalian circadian oscillator is the suprachiasmatic nucleus (SCN) in the hypothalamus. The hypothalamus controls a number of important physiological functions, such as feeding and drinking, that are also abnormal in R6/2 mice [32], [33]. This may be caused by hypothalamic neuronal degeneration/atrophy [34]. However, although the circadian rhythms of R6/2 mice are disrupted, the SCN itself appears to function normally *in vitro* [16]. This suggests that the abnormal behavioural and molecular circadian rhythms observed in R6/2 mice arise from dysfunction of brain circuitry afferent to the SCN, rather than the pacemaker itself [16]. Although we could not measure circadian rhythms directly in this experiment, we hypothesize that disruptions to the sleep-wake patterns of these mice may have resulted from enrichment during circadian day. These disruptions, together with developing deficits in metabolism [32] and motor function [35], might have left the R6/2 mice increasingly more tired and less rousable than their WT littermates. It is possible, therefore, that the increasing inactivity seen in the R6/2 mice was a result of decreased strength and energy. This hypothesis is supported further by the body weight data from this study, which showed weight loss in all R6/2 mice by the end of the enrichment period. It is interesting that there was a tendency among R6/2 home cage mice of both sexes for the no-handling groups to be heavier than handling groups towards the end of the enrichment period. Interestingly, in the male R6/2 handling groups, playground exposure led to increased body weight at the end of the enrichment period, a tendency that was not observed in female groups. These findings further support a sex-specific mix of beneficial and detrimental effects of the two types of enrichment.

Sex-dependent differences in normal behaviour have been reported in other rodent models of HD. In a rat model, male animals display increased daytime activity at an earlier stage of phenotype than female rats [36]. In the N171-82Q model, male mice show poorer performance on the rotarod than female mice [37]. In the YAC128 model, female mice live longer than male mice [38]. A detailed examination of the 140 CAG knock-in model of HD also revealed a number of sex differences, including increased grooming and dark phase running in female mice, and decreased climbing in male mice [39]. These studies have shown sex differences in phenotypically-altered behaviours, but ours is the first to demonstrate that modulation of the environment also has sex-dependent effects in R6/2 mice, with enrichment having either positive or negative effects depending upon the sex of the mouse.

One unexpected finding is the trend towards changes in body weights in WT mice, long after the end of the EE period. Male WT mice that had been exposed to the playgrounds tended to have higher weights than home cage mice (in both the handling and no handling conditions) from around 21 weeks of age, although this difference reached significance in the handling group only. It is unclear what could have caused this, since by the time the effect developed, the mice had been out of the playgrounds for 6 weeks.

Results from this experiment have demonstrated clearly sex differences in response to EE. While exposure to the playgrounds, for 6 hours a day over a 6 week period, significantly improved survival in both male and female R6/2 mice, beneficial effects of EE on cognition were seen in females only. Although the effects we observed were not as marked as those reported in other EE studies, there are two possible reasons for this. The first reason is that we used R6/2 mice, which have an early, aggressive onset of phenotype, and so have been rarely used in EE experiments. The second reason is that our mice are routinely kept in conditions that would, in most labs, be considered “enriched” already. They are group-housed, have plastic nest boxes, a range of bedding, lowered water bottle spouts, and a mashed food supplement to facilitate feeding and help maintain hydration. This home cage enrichment raises the threshold for beneficial changes and makes them less likely. Data from the current study, which show that enhanced EE can produce a further improvement in cognitive ability and survival, are very encouraging in the context of using EE to improve the quality of life of HD patients.

## Supporting Information

Figure S1 Quadrant and zone preferences during first Morris water maze (MWM) trial. Percentage times spent in each quadrant (A) or zone (B) during the first training trial in the MWM are shown for WT and R6/2 mice, with data from both sexes and all experimental groups pooled. All data shown are mean ± s.e.m. n.s.  =  non-significant, * p<0.05, ** p<0.01, *** p<0.001.
(0.30 MB TIF)

Movie S1 Example of playground configuration.
(4.03 MB MP4)

Movie S2 Demonstration of the gentle handling used to keep mice active.
(1.60 MB MP4)
