# Correction: Responses to Environmental Enrichment Differ with Sex and Genotype in a Transgenic Mouse Model of Huntington's Disease

**DOI:** 10.1371/annotation/4a16d713-6dd6-4366-96a8-71d316456f98

**Published:** 2010-09-01

**Authors:** Nigel I. Wood, Valentina Carta, Stefan Milde, Elizabeth A. Skillings, Catherine J. McAllister, Y.L. Mabel Ang, Alasdair Duguid, Nadeev Wijesuriya, Samira Mohd Afzal, Joe X. Fernandes, T.W. Leong, A. Jennifer Morton

The 12th author's name was incorrect as published. The correct name is A. Jennifer Morton.

