# Supplementary figures and images for: Responses to Environmental Enrichment Differ with Sex and Genotype in a Transgenic Mouse Model of Huntington's Disease
Source: PLoS One. 2010 Feb 12;5(2):e9077. doi: 10.1371/journal.pone.0009077 (PMC2820540; doi:10.1371/journal.pone.0009077)

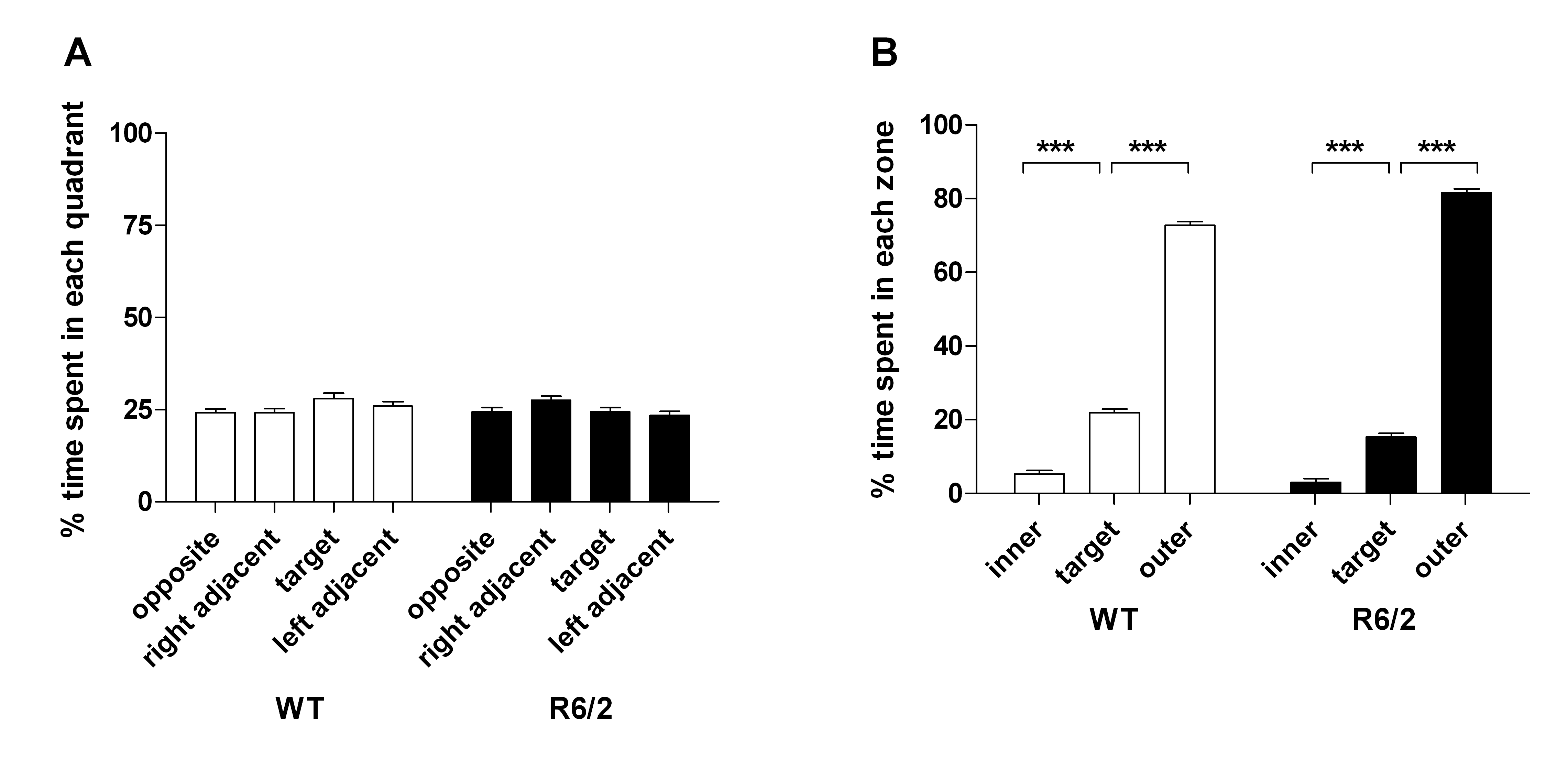

Supplement: Figure S1 — Quadrant and zone preferences during first Morris water maze (MWM) trial. Percentage times spent in each quadrant (A) or zone (B) during the first training trial in the MWM are shown for WT and R6/2 mice, with data from both sexes and all experimental groups pooled. All data shown are mean ± s.e.m. n.s. = non-significant, * p<0.05, ** p<0.01, *** p<0.001. (0.30 MB TIF) [file pone.0009077.s001.tif]
